# A Student-Led Community Outreach Telehealth Program for COVID Education and Health Promotion (COACH)

**DOI:** 10.1093/geroni/igab046.3455

**Published:** 2021-12-17

**Authors:** Michelle Yang, Cam Clayton, Devin Harris, Chelsea Pelletier, Julia Schmidt, Jill Zwicker, Brodie Sakakibara

**Affiliations:** 1 University of British Columbia, Richmond, British Columbia, Canada; 2 University of British Columbia, Vancouver, British Columbia, Canada; 3 Interior Health Authority, Kelowna, British Columbia, Canada; 4 University of Northern British Columbia, Prince George, British Columbia, Canada; 5 University of British Columbia, Kelowna, British Columbia, Canada

## Abstract

While public health measures of quarantining, socially isolating, and physical distancing are important to minimize the spread of coronavirus (COVID-19), these actions may also compromise the ability to manage one’s own health, thereby increasing the risk of adverse health events. The purpose of this study was to evaluate a student-delivered Community Outreach teleheAlth program for Covid education and Health promotion (COACH) to community-living adults (age ≥65 years). We hypothesized that COACH would improve health promoting behaviour as measured by the Health Directed Behaviour subscale of the Health Education Impact Questionnaire. We also anticipated COACH would improve secondary outcomes of perceived stress, depressive and anxiety (Depression, Anxiety, and Stress Scale-21), social support (Medical Outcomes Study Social Support Survey), health-related quality of life (Short Form-36), and health promotion self-efficacy (Self-Rated Abilities for Health Practices Scale). In this single-group pre-post study, we recruited 75 community-living adults with access to telephone/video-conferencing technology to participate in six 30-45 minute sessions with trained medical students over a two-month period. The mean age of participants was 72.4 years (58.7% female), with 80% reporting two or more chronic conditions. No participants were diagnosed with COVID-19 during participation. Paired sample t-tests showed significant improvement in health directed behaviour (p < .001, d = 0.45) and self-efficacy (p <.001, d = 0.44), but significant decrease in mental health-related quality of life (p < .001, d = -1.69). Overall, COACH may help improve health directed behaviour and health promotion self-efficacy, despite decreases in mental health possibly associated with COVID-19 restrictions.

